# Evaluation of the frequency of attention deficit hyperactivity disorder in patients with asthma

**DOI:** 10.1186/s12948-023-00185-4

**Published:** 2023-06-27

**Authors:** Matineh Nirouei, Mona Kouchekali, Homa Sadri, Mostafa Qorbani, Hadi Montazerlotfelahi, Narges Eslami, Marzieh Tavakol

**Affiliations:** 1 Alborz University of Medical Sciences, Karaj, Iran; 2 Non-Communicable Diseases Research Center, Alborz University of Medical Sciences, Karaj, Iran; 3 Department of Pediatrics, Imam Ali Hospital, Alborz University of Medical Sciences, Karaj, Iran; 4 Department of Pediatric Neurology, Imam Ali Hospital, Faculty of Medicine, Alborz University of Medical Sciences, Karaj, Iran; 5 Department of Allergy and Clinical Immunology, Mofid Hospital, Shahid Beheshti University of Medical Sciences, Tehran, Iran

**Keywords:** ADHD, Allergy, Asthma, Karaj

## Abstract

**Background:**

Asthma is the most prevalent respiratory disease caused by chronic airway inflammation. Attention Deficit Hyperactivity Disorder (ADHD) is children's most common psychological and neurodevelopmental disorder. Increased risk for ADHD in patients with inflammatory and autoimmune diseases supports the role of inflammatory mechanisms in the occurrence of ADHD. However, the association between asthma and ADHD remains unclear.

**Objective:**

This study was designed to evaluate the prevalence of ADHD in patients with asthma who were referred to the clinic of allergy and clinical immunology.

**Methods:**

This cross-sectional study was conducted on children aged 6 to 18 with asthma at Imam Ali hospital, Karaj, Iran. The patient's demographic data, history of childbirth delivery type, premature birth, hospital admission, family income, birth rate, and family history information related to the patient's asthma and medicines were recorded. ADHD diagnosis was made using the Persian version of Conners Parent Behavioral Problems Rating Scale (CPRS-26).

**Results:**

In this study, 677 asthmatic patients were enrolled; 46 patients (6.8%) had ADHD. The probability of ADHD in asthmatic patients inhabited in a rural area, males, and patients with a history of food allergy, allergic rhinitis, urticaria, and eczema was significantly higher (p < 0.05). In addition, our result demonstrated that the likelihood of ADHD in patients with asthma and a history of PICU admission was significantly higher (p < 0.05).

**Conclusions:**

The present study showed that severe asthma, was the risk factor for ADHD in patients with asthma. Physicians should be aware of this co-morbidity to refer asthmatic patients who have the symptoms of ADHD to a psychologist.

## Background

Asthma is one of the most common chronic inflammatory diseases with onset in childhood. The four main asthma symptoms are recurrent cough, wheezing, shortness of breath, and tightness in the chest [1, 2]. It is a chronic inflammatory disorder due to the interaction between environmental and genetic factors, presented with reversible constriction of the airways [3].

Attention deficit hyperactivity disorder (ADHD) is the most common behavioral and neurodevelopmental disorder in children, affecting many aspects of a patient's life [4]. ADHD is characterized by age-inappropriate and impairing levels of inattention, hyperactivity, and impulsivity, which cause impaired learning abilities, sleep disorder, and social isolation; therefore, it reduces patients' and families' quality of life [5, 6].

Detection of increased risk for ADHD in patients with inflammatory and autoimmune diseases, inflammation-associated genes, and studies on markers associated with inflammation support the role of inflammatory mechanisms in the occurrence of ADHD [7]. The effect of inflammatory cytokines might explain this. For instance, it has been reported that increased IL-6 levels may change neural pathways of the developing brain and affect attention and memory with its effect on neurogenesis and synaptic plasticity in the prefrontal cortex and hippocampus [8]. Recent studies suggested that ADHD patients are more likely to have allergy-associated disorders, including asthma, allergic rhinitis, allergic conjunctivitis, atopic dermatitis, and psoriasis [9, 10].

The prevalence of ADHD in patients with asthma was assessed in a recent meta-analysis. All studies that concerned the association between asthma and ADHD were enrolled. After applying the inclusion and exclusion criteria, 84 out of 2815 articles fulfilled the principles, which included 49 datasets. ADHD was seen in 210,363 participants, contrary to 3,115,168 non-ADHD people. Asthma was found in 16.9% and 11_·_5% of individuals with and without ADHD, respectively. The authors included a Swedish population-based cohort study with 1,575,377 individuals, of whom 259,253 (16·5%) had asthma and 57,957 (3·7%) had ADHD. They reported that the prevalence of ADHD was significantly higher in individuals with asthma than those without (p < 0.0001). According to this study, the overall prevalence of ADHD in subjects with and without asthma was 8. 8% and 5.6%, respectively [11].

Some case reports of altered behavior and psychosis in children who used ICS (inhaled corticosteroids) have been reported. It suggested the hypothesis that the link between asthma and ADHD may be because of asthma medication such as inhaled corticosteroids [12]. However, larger studies indicated that there was no strong evidence that the standard treatment of asthma causes ADHD [13].

## Objectives

ADHD disorder has a complicated diagnosis, treatment, and follow-up process, which imposes a great economic and psychological burden on society and the family. It has been proposed that children with asthma could have behavioral diseases such as ADHD more than other children [14, 15]. Most studies have shown that the co-existence of asthma in ADHD patients increases the risk of hyperactivity and impulsivity symptoms [2, 16]. Thus, we decided to determine the prevalence of ADHD in asthmatic patients.

## Methods

### Study design

This cross-sectional study enrolled asthmatic patients aged between 6 and 18 years old. Six hundred seventy-seven unrelated cases with asthma referred to the clinic of allergy and clinical immunology of Imam Ali hospital, Karaj, Iran, were enrolled. The clinical diagnosis of asthma in this study was done by an allergist based on Global Initiative for Asthma (GINA) 2017 criteria, which included a typical history of variable respiratory symptoms such as wheezing, chest tightness, shortness of breath, and coughing. Then, variable expiratory airflow limitation was confirmed by one or more of these tests: positive bronchodilator reversibility test which means increase in Forced expiratory volume in the first second (FEV1) more than 10–12% predicted or positive exercise challenge which means decrease in FEV1 of more than 10–12% predicted or peak expiratory flow (PEF) of more than 15% [17].

Exclusion criteria were as follows: neurodevelopmental delay, epilepsy, cerebral palsy, mental retardation (which was approved by a pediatric neurologist), history of using anti-epileptic drugs.

### Sample size

The prevalence of ADHD among asthmatic children is 7%, with a confidence interval of 95% and a maximum acceptable error of 2%; in this study, the sample size of 10% drop in person estimated 685 patients.

$${\text{N}} = {\text{ z2pq}}/{\text{d2}}{.}$$

### Data extraction

Demographic and background information of the patients was collected based on a questionnaire designed by the researcher. The hyperactivity-attention deficit status of the patients was evaluated using the Persian version of the Conners Parent Behavioral Problems Rating Scale (CPRS-26). This questionnaire is one of the standard and well-known tools for measuring children's hyperactivity attention deficit, which identifies five factors including learning, psychosomatic, and behavioral problems, as well as impulsivity-hyperactivity, and anxiety. It includes 26 questions, graded according to the severity of symptoms with a Likert scale of completely true = 4, relatively true = 3, somewhat true = 2, and never = 1 [18].

The reliability and validity of this scale have been confirmed in several studies. The reliability and validity of the Persian version of this questionnaire were reported to be 90% and 85%, respectively [19, 20].

Demographic information comprising a comprehensive medical history including age, gender, childbirth delivery type, premature birth, hospital admission, birth rate, atopic dermatitis, allergic rhinitis, food allergy, family income, as well as data concerning patient's asthma including the age of diagnosis, asthma medications and family history of asthma, parent's education, and parent's job were recorded.

### Statistical analysis

Information was collected in questionnaire sheets and was analyzed using SPSS statistical software version 25.0 (IBM corporation, Chicago, IL, USA). Qualitative variables were reported as percentage and frequency. Chi-square test was used to analyze the correlation between qualitative variables. Quantities variables analyzed with T-Test or Mann–Whitney (based on parametric or non-parametric).

## Result

### Demographic data

A total of 677 children with asthma (58.9% males and 41.1% females) with a median current age of 11.78 ± 2.98 years old were enrolled; among whom 46 patients (6.8%) were diagnosed with ADHD.

### Comparison of demographic information between asthmatic patients with and without ADHD

The mean age of patients with ADHD was 11.02 ± 2.71 years old, whereas the mean age of patients without ADHD was 11.84 ± 2.9 years old (p value: 0.061). Regarding gender, 34 out of 46 (73.9%) patients with asthma and ADHD were male, whereas, among 631 patients without ADHD, 365 (57.8%) were male. The results showed a significant gender difference in the association between asthma and ADHD (p value < 0.05). Also, 4.8% of patients with ADHD lived in rural areas, whereas 13% of patients without ADHD lived in rural areas. This indicated that ADHD was significantly related to the place of living (p value < 0.05). In other words, asthmatic patients who lived in rural areas were significantly more likely to be suffering from concomitant ADHD. There was no significant association between parent’s education with ADHD disease (Table 1 represented father’s, and mother’s education in patients with ADHD and patients without ADHD).

**Table 1:** Represented father’s and mother’s education in patients with ADHD and patients without ADHD

Education	With ADHD	Without ADHD	p value
Father’s education			
Below diploma	10 (21.7%)	180 (28.5%)	0.546
Diploma	22 (47.8%)	245 (38.8%)	
Bachler	6 (13%)	118 (18.7%)	
Master	6 (13%)	58 (9.2%)	
PHD	2 (4.3%)	30 (4.8%)	
Total	46 (100%)	631 (100%)	
Mother’s education			
Below diploma	11 (23.9%)	134 (21.2%0	0.449
Diploma	25 (54.3%)	341 (54%)	
Bachler	9 (19.6%)	102 (16.2%)	
Master	1 (2.2%)	54 (8.6%)	
PHD	0 (0%)	0 (0%)	
Total	46 (100%)	631 (100%)	

### Comparison of past medical history between asthmatic patients with and without ADHD

Concerning the type of delivery, 50% of patients with ADHD and 46.1% without ADHD delivered vaginally, which showed no significant difference between the type of delivery (caesarian vs. vaginal) and ADHD in asthmatic patients. The patients born by a cesarean were divided into three groups based on the reason for cesarean: mother desire, obstetrics indication, and other reasons. Cesarean in patients without ADHD was done in 128 patients for their mother's wish, 95 patients for obstetrics indications, and 117 patients for different reasons. In addition, 12 patients with ADHD were born caesarian because of their mother's desire, six patients for obstetrics indications, and five patients with other reasons. There was a significant difference between the two groups (p value > 0.05). The duration of asthma symptoms in patients with and without ADHD was 6.73 ± 2.04 years and 7.03 ± 2.02 years, respectively. No considerable distinction was found between patients with and without ADHD regarding the years of suffering from asthma. There was no meaningful correlation between suffering from ADHD and a history of hospitalization because of an asthma attack. The detailed comparison of two study groups regarding demographic data and past medical history are illustrated in Table 2.

**Table 2:** Represented detailed past medical history of patients with ADHD and patients without ADHD

Variable	With ADHD (n = 46)	Without ADHD (n = 631)	p value
Consanguinity			
Yes	17 (37%)	205 (32.5%)	0.520
No	29 (63%)	426 (67.5%)	
NICU admit			
Yes	4 (8.7%)	53 (8.4%)	0.688
No	42 (91.3%)	578 (91.6%)	
Mechanical ventilation			
Yes	2 (4.3%0)	38 (4%)	0.763
No	44 (95.7%)	593 (64%)	
Preterm labor			
Yes	9 (19.6%)	119 (18.9%)	0.847
No	37 (80.4%)	512 (81.1%)	
Second hand smoke exposure			
Yes	17 (37%)	243 (38.5%)	0.877
No	29 (63%)	388 (61.5%)	
Hospitalization due to asthma attack			
Yes	18 (32.1%)	299 (47.4%)	0.093
No	28 (60.9%)	332 (52.6%)	
PICU			
Yes	10 (21.7%)	67 (10.6%)	0.030*
No	36 (78.3%)	564 (89.4%)	
Tonsillectomy			
Yes	5 (10.9%)	58 (9.2%)	0.606
No	41 (89.1%)	573 (90.8%)	
Asthma family history			
Yes	32 (69.6%)	397 (62.9%)	0.430
No	14 (30.4%)	234 (37.1%)	
Food allergy			
Yes	12 (26.1%)	88 (13.9%)	0.032*
No	34 (73.9%)	543 (86.1%)	
Allergic rhinitis history			
Yes	21 (45.7%)	181 (28.7%)	0.019*
No	25 (54.3%)	450 (71.3%)	
Urticaria/ eczema history			
Yes	17 (37%)	144 (22.8%)	0.046*
No	29 (63%)	487 (77.2%)	

*p value  significant (<0.05) shown in bold

The mean number of PICU admission was significantly different between the two groups. Patients with ADHD were 2.10 ± 0.99 times admitted to PICU, whereas patients without ADHD were admitted to PICU 1.19 ± 0.60 times (p value: 0.0001).

### Comparison of medications between asthmatic patients with and without ADHD

There was no significant difference between the two groups of patients in the type of medicines they received, representing the level of asthma severity and the relationship between ADHD and a specific medication (Table 3).

**Table 3:** Represented detailed treatment in patients with ADHD and patients without ADHD

Treatment	With ADHD (n = 46)	Without ADHD (n = 631)	p value
Using salbutamol spray			
Always	34 (73.9%)	475 (75.3%)	0.830
During exercise	1 (2.2%)	12 (1.9%)	
In a cold weather	9 (19.6%)	103 (16.3%)	
During dyspnea	2 (4.3%)	41 (6.5%)	
Type of spray			
Fluticasone	19 (41.3%)	285 (45.3%)	0.421
Combination^a^	14 (30.4%)	222 (35.2%)	
Salbutamol	12 (26.1%)	104 (16.5%)	
Other	1 (2.2%)	19 (3%)	
Type of drug			
None	13 (28.3%)	183 (29%)	0.646
Anti-leukotriene	21 (45.7%)	313 (49.6%)	
Antihistamine	6 (13%)	85 (13.5%)	
Corticosteroid	6 (13%)	50 (7.9%)	

^a^Combination: combination of inhaled steroid and long-acting bronchodilator inhaler

## Discussion

The frequency of attention deficit hyperactivity disorder (ADHD) in children with asthma who were referred to the clinic of allergy and clinical immunology of Imam Ali hospital was evaluated in this study. The study group included 677 patients with asthma; among them, 46 patients (6.8%) had ADHD, which was diagnosed using the Persian version of the Conner’s Adult ADHD Rating Scale questionnaire.

This survey showed that the male–female ratio was significantly higher in the group with ADHD than in patients without ADHD. Mu-Hong Chen studied 14,812 atopic subjects, including 9438 (63.7%) with asthma, and in comparison, they studied 6944 non-atopic subjects as a control group. All subjects were followed until the onset of events (ASD or ADHD). They reported that patients with asthma were at a higher risk of ADHD (p-value: 0.001). In addition, the male gender was an independent risk factor for ADHD in asthmatic patients [21]. It might be because of the gender difference in ADHD itself, regardless of being associated with asthma or not.

In this study, the history of PICU admission due to asthma attack in patients with ADHD was significantly higher than in patients without ADHD. PICU admission indicates the severity of asthma, so it is possible to consider the hypothesis that severe asthma is a risk factor for ADHD. In addition, the average number of hospitalizations in PICU due to asthma attack in patients with ADHD was significantly higher than in patients without ADHD. According to the results obtained about the severity of asthma and ADHD, we suggest that patients with severe asthma could release inflammatory cytokines more than others [7, 22]. Therefore, severe asthma might cause a higher risk of ADHD. In previous studies, it has been shown that the Inflammation state affects the development of both ADHD and asthma [3]. Holmberg et al. in a population-based cross-sectional study including 20,072 twins in the Swedish nation, proposed that the severity of asthma might be a risk factor for ADHD in asthmatic children [23]. Yossy Machluf et al., in a cross-sectional study of 113,671 Israeli adolescents (including 66,547 males and 47,124 females), reported associations between mild and moderate-to-severe asthma phenotypes and coexistent medical conditions like ADHD within each gender separately [24].

There was no considerable correlation between types of medications in the present survey. In line with our findings,  Holmberg et al. reported that asthma medications (inhaled b2-agonists and inhaled corticosteroids ≥ 3 times) didn't increase the risk of ADHD [23]. Also, Po-Yu Huang et al., in their population-based cohort study published in 2021, evaluated 54,487 asthmatic children from the Taiwanese nation. They reported that montelukast didn't increase the risk of ADHD among asthmatic patients, which is consistent with the results of the present study [25].

In the present study, the history of allergic rhinitis was significantly higher in patients with ADHD (p-value: 0.019), which concord with the result of the previous studies [26, 27]. Ho Jang Kwon et al. studied 549 children with ADHD and a control group of 3564 children. They reported a significant association between ADHD, childhood asthma, and allergic rhinitis among Korean children [28].

Previous studies demonstrated a relationship between ADHD and allergic diseases like asthma, allergic rhinitis, urticaria and eczema, and food allergies, which concord with the present study [29–32]. In addition, our result showed that a history of food allergies and a history of urticaria and eczema were significantly higher in the patients with ADHD compared to those without ADHD [31, 32]. It may confirm the hypothesis that releasing inflammatory mediators and allergies can cause ADHD.

Anyan Huang et al., based on a meta-analysis of 6663 ADHD patients and 93,825 controls published in 2020, suggested that second-hand cigarette smoke exposure may be a risk factor for ADHD. In contrast, this study didn't find any relationship between ADHD and being a second-hand smoker. It might be explained by the higher number of their study population [33].

Aslıhan Yüksel et al. studied children aged 6 to 14, and they evaluated 91 cases with ADHD and 264 without any psychopathology. They reported that tonsillectomy did not predict ADHD, which concurs with this study [34].

In this study, there was no significant relationship between the history of preterm labor in patients with and without ADHD. Whereas, in the previous studies, like a Finnish population-based study on 10,321 ADHD patients and 38,355 controls, a history of preterm labor was found to be a risk factor for ADHD in children [35]. This difference might be due to the fewer number of patients that were included in our study.

## Conclusion

Considering the fact that both ADHD and asthma are amongst the most frequent health problems, the findings of this study, which showed the co-existence of these disorders and evaluated the related risk factors, seem to be worthwhile. This co-morbidity should be taken into account by physicians for timely diagnosis and referring the asthmatic patients who have ADHD symptoms to a psychologist.

## Data Availability

All data generated or analyzed during this study are included in this published article.
